# Lifting the veil on Bohm’s holomovement

**DOI:** 10.1080/19420889.2021.2001157

**Published:** 2021-11-22

**Authors:** Andrew Lohrey, Bruce Boreham

**Keywords:** Holomovement, wholeness, nonlocality, local realism, meaning, consciousness, worldviews

## Abstract

In this paper, we argue that Bohm’s unbroken and undivided totality he called the holomovement, the title he gave to the concept of the self-organizing universe, is more coherently understood when viewed as universal consciousness. Bohm’s understanding of consciousness oscillates around being a quality of local minds and the interconnected totality of the holomovement. We suggest such equivocations impose limitations on Bohm’s general holistic framework because they import into his model the limiting restrictions of Cartesian separation and are, therefore, incongruous for use within his holistic model of the holomovement. We also argue that the term ‘meaning’ has a structural and functional agency appropriate to Bohm’s model of the holomovement, while also reflecting the living characteristics of this organic totality that is full of meaning.

## Introduction

David Bohm’s friend and colleague, Basil J. Hiley wrote, **‘**I have always felt that wholeness was the key to understanding quantum phenomena.’ Hiley went on, ‘In this regard the Bohm model has served its purpose. It has shown that it is possible to lift the veil of reality, but has it been lifted far enough’? [1, p.7] This is the question we ask in this paper in relation to the reality of wholeness, has the veil of reductionism been completely lifted from Bohm’s model of wholeness and if not, what would the scope and contours of a coherent model of wholeness look like?

*Wholeness and the Implicate Order* [2], was the seminal book by David Bohm (1917–1992) that introduced his model of wholeness. In this book, Bohm began with a criticism of Cartesian dualism as it applied to relativity theory and to quantum physics, commenting that the Cartesian order is ‘leading to serious contradictions and confusion’ [2, p.xv]. His positive response to the Cartesian fragmentation of knowledge was a new order that had its roots in the experiments of quantum physics, and which he said was appropriate to a universe of unbroken wholeness. A ‘universe of unbroken wholeness’ represented a world view quite distinct from the Cartesian dualist world view that has at its heart the separation of the mental from the physical, a dualism that is commonly phrased as subjectivity versus objectivity.

To be coherent, a world view that proclaims the universe as an unbroken wholeness, must be applicable to the whole, that is, to everything without exception. In other words, its application should be universal and that will include not just the field of physics but also of biology and psychology. That means not simply wholeness in relation to the non-organic, but also wholeness in relation to organic life and the Singularity of Nature (that is, the way that nature appears to mesh interconnectedly) [3] as expressed by Torday and Miller [3]. In a related paper, these authors [4] propose that the quantum principles of non-locality, entanglement and quantum coherences are active biological mechanisms and that life on earth has been dependent on quantum processes from its earliest beginnings. Quantum coherence and entanglement have been shown to be the active operating means of excitation of the light harvesting complexes used by photosynthetic bacteria. In addition, the avian magnetic compass is dependent on quantum superposition and the quantum entanglement of particles, while cell–cell communication extends across an entire organism by a variety of quantum means. These authors conclude that the interrelationship between the physical and biological realms is an established feature of ongoing processes.

Yet importantly, the question of a coherent wholeness must also include those ‘stateless subjects’ – meaning, mind, language and consciousness – that have been continually turned back in their attempts to cross the Cartesian sea of separation and division by formal exclusions that try to prevent them from arriving at the ports of mainstream science. In this paper, it is the refugee subjects of meaning and consciousness that we focus on in regard to Bohm’s view of wholeness. The reason for this emphasis is Bohm’s own interest in and concern with both these subject matters and how they relate to his model of wholeness.

Bohm’s view of wholeness appears to have come from two quarters; firstly, as a reaction to the severe limitations of Cartesian dualism and its deleterious effects on science; and secondly as a broad interpretation that has grown out of research and experiments in quantum science. What then are the characteristics of Bohm’s model of wholeness? He identified these characteristics by using the example of a hologram as a way of describing the interactive nature of undivided wholeness. Yet he also produced his own theory of wholeness in *Wholeness and the Implicate Order*. We take the example of the hologram first.

A hologram is created when coherent laser light is reflected (or scattered) from an object and collected on a photographic plate along with part of the original laser beam. The two beams interfere to generate a standing wave pattern which is recorded directly on the plate. The intensity on the photographic plate is the square of the sum of the amplitudes of the reflected (or scattered) light and the reference beam. After development, the photographic plate can be illuminated with a laser beam that interacts with the interference pattern to produce a three-dimensional image that looks like the original object, but this image appears to us if we are looking through a window.

The significant feature of the hologram that Bohm focussed on was the complex relationship between the parts and the whole. In a hologram the local regions of the original object are mapped into every region of the hologram and if a laser beam is used to illuminate only a small fragment of the photographic plate, we do not see a fragment of the image but instead, we see the whole image in somewhat less sharply defined detail. What does this tell us about the interactive and wholeness character of the hologram? It tells us that each fragment of the image is not only a part of the whole but also it is an instance of the whole. That means that the whole of the hologram inheres in or is immanent within each fragment or part while each part contributes to the whole.

These interactive relationships between parts and whole that are produced by coherent light in holograms can be called symmetrical. That means these holographic relationships can be detailed as: ‘whole-to-part/part to whole’ relationships. In addition, the unifying force that holds the parts and the whole together is structured by a complex of whole-to-part/part-to-whole relations that exhibits a symmetrical force or unity. This force creates the indivisible unity of the hologram so that the parts, while still distinct are locked together into the whole image in ways that cannot be separated or divided into separate or linear series. Bohm used the example of the hologram to discuss the universal nature of wholeness in that each region of space: ‘the movement of light implicitly contains a vast range of distinctions of order and measure, appropriate to a whole illuminated structure. Indeed, in principle, this structure extends over the whole universe and over the whole past, with implications for the whole future’. [2, p.148]

Hence, the example of a hologram became an interactive model having a scope that takes in the whole of space and time of the universe. In this sense, his wholeness model does incorporate everything in the universe. But Bohm also developed his own theory of universal wholeness which was supported by the interactive character of the hologram. This was his theory of the implicate and explicate orders. The explicate order represents the explicit differentials forms and objects (the parts) that move within of the physical world of space and time and which we will now say, arise through the local mind’s processes of perception. In contrast, Bohm’s implicate order represents the entire universal context of a singular, whole and unified interconnected system – an undivided universe. The entire implicate order can be described as having unifying qualities but devoid of quantities, rather quantities are the central feature of the explicate order.

The universe wide unifying context of the implicate order is entirely implicit, a term that Bohm suggests is based on the verb ‘to implicate’, which means ‘to fold inwards’. [2, p.149] He speculated that each region of space and time ‘contain a total structure ‘enfolded’ within it’. Hence, the implicate order can be described as an enfolding order while in contrast, the explicate order unfolds from the implicate order the moving forms and objects of the explicit and physical universe. This means that all physical objects and forms observed to be moving in the continuum of space and time represent the explicate order, which unfolds from the implicate order. From a Cartesian perspective, the explicate order is the first and only order of importance.

The relationship between Bohm’s two orders is not dualistic but highly integrated in that the explicate order arises out of the movements of the implicate order and together these two orders produce an undivided universe, which is a wholeness where there are no separations or gaps. At times Bohm refers to the totality of this wholeness as the ‘holomovement’. [2, p.178; 5, p.273] As a consequence of this totality, Bohm suggests that ‘everything is to be explained in terms of forms derived from this holomovement’. [2, p.178] The clear implication of this statement is that the holomovement arranges, organizes and exerts agency over the derived forms of the explicate order – the visible and physical universe.

In his paper, *Quantum Reality Unveiled Through Process and the Implicate Order* [1] Hiley describes some of the properties of wholeness in regard to quantum physics by suggesting that ‘what underlies all material structures and form is the notion of activity, movement or process.’ [1, p.9] This background of continual change represents pure activity or flux in which all matter and substance are seen as explicit, semi-autonomous, quasi-local invariant features of the background movement. Hiley writes that Bohm described this fundamental background flux as ‘movement’ and the entire fundamental background as the holomovement. It appears to us that the continual background flux of the holomovement represents the results of the agency of this totality. In addition, to be coherent the totality of the holomovement must represent a singular system that contains nested sub-systems. In other words, while there may be a diversity of wholes in physics and biology there is only one over-riding wholeness that connects all these sub-systems together. As Bohm and Hiley state in *The Undivided Universe*, ‘The essential features of the implicate order are, as we have seen, that the whole universe is in some way enfolded in everything and that each thing is enfolded in the whole’. [6, p.382]

The agency of the holomovement is also implied in the term ‘order’ that Bohm uses to describe his model of wholeness. The term ‘order’ implies an arrangement of parts that has been already predetermined through a pre-established set of conditions inherent in the holomovement. Hence, the ‘implicate order’ is an order that not only enfolds everything, (and everything means from particles to molecules to organism to galaxies) but also this primary order contains the organizing potentials and agency out of which all forms and objects arise. ‘All things found in the explicate order emerge from the holomovement and ultimately fall back into it’. [6, p.382] Hence, the primary unifying agency in the universe is thus contained within the potential forces of Bohm’s holomovement. In other words, there is only one whole in the universe and that is the current singularity of the holomovement.

This conclusion is reinforced by the definition that Hiley provides in his 2008 paper where he describes wholeness like this: ‘As we are so immersed in reductionism, it is very difficult to know what the notion of wholeness actually means. Put simply wholeness implies that the properties of the individual parts are determined by the order of whole, rather than the parts determining the whole’. [1, p.7] Hiley’s definition of wholeness turns the traditional Cartesian approach, focussed on locality and determinism on its head by proposing that the holomovement’s overall agency is determined by the conditions of the nonlocal whole and not by the local parts. As the organizing agency of parts is traditionally seen as a local agency, so too the agency of the whole must represent a nonlocal agency. The order of the whole can thus be called a nonlocal order and following Bohm’s model in another place we have called this nonlocal order, ‘The Nonlocal Universe’ [7].

In addition, Bohm’s model of the implicate and explicate orders reverses the Cartesian’s local fixation on physical forms and objects and this is made explicit by his proposition, ‘that in the formulation of the laws of physics, primary relevance is to be given to the implicate order’. [2, p.150] Applying this proposition means giving primary relevance to the unity and connections that the holomovement provides. Hence, in terms of the unifying agency of the holomovement the individual parts of the universe (physical objects and forms) come into existence and their movements are determined by the organization and order of the whole. Yet even while acknowledging the primary agency of the holomovement what is left unstated by both Hiley and Bohm is the question of the conditions of the ‘order of the whole’. What does that mean in terms of the whole’s structure and function? Has the veil been lifted far enough?

In order to reflect on that question, we need to go back to the genesis of Bohm’s implicate order, which are the conditions of meaning. As already referred to, Bohm’s focus on the implicate order derived from the term ‘implicit’ which he suggested is based on the verb ‘to implicate’ or to fold inwards. While this may be correct, the term ‘implicit’ primarily relates to a major condition of meaning. With his interpretation of ‘enfolding’ Bohm also provides a physical example of enfolding with the ‘unmixing experiment’ that involved two concentric transparent cylinders that rotate relative to each other. This is an experiment referred to by both Bohm [2, p.154] and Hiley [1, p.17] and is interesting because it is entirely physical. The impression the reader receives from the descriptions of this experiment is that enfolding, and unfolding are physical processes.

While that experiment does indicate there can be physical processes of enfolding and unfolding, we should not forget that these critical terms refer back to the functions of meaning and as meaning represents a metaphysical state, the terms’ enfolding’ and ‘unfolding’ represent metaphysical functions of meaning. In terms of the structure and function of meaning, ‘unfolding’ stands as an approximate term for the general transformations that unfold explicit meaning (objects, forms and differences) from their implicit contexts, while enfolding represents the reverse transformation. Bohm also used the term ‘enfolding’ in a more general sense of a field that enfolds forms and objects as we shall see in the next section in relation to energy and matter. What then are the conditions of meaning and how do these conditions relate to the holomovement?

## Meaning

Meaning is a subject matter that over the years a range of European philosophers and linguists have directed their attention toward. Many of these have understood meaning to be the by-product of signs, language, mind or intentionality (Brentano, Saussure, Pierce, Ogden and Richards, Wittgenstein). Others like the phenomenologists have sought to tie meaning to the conscious experience of phenomena (Husserl). The authors of this paper have a forty-plus year history of researching, writing and analyzing the subject matter of meaning and that work has manifest in a range of books on the subject, the last two being Lohrey, 2018, 2020. Perhaps the author that has influenced our theory of meaning to a large degree is David Bohm with his book, *Wholeness and the Implicate Order* [2].

How is it possible for Bohm, a physicist writing about quantum physics to have any influence on a metaphysical theory of meaning? We consider that his influence is an indication of the completeness of his research and the coherence of his theory of wholeness. We suggest that for a theory of wholeness to be so, even if it is directed at the physical world, it would have to include in some manner the central role of meaning in human experience. Bohm’s theory of the implicate and explicate orders rests on the metaphysical conditions of meaning, that is, on the nature of implicit and explicit meaning. Hence, our theory concerning the wholeness of meaning reflects to a large degree Bohm’s theory of wholeness.

In essence, Bohm’s theory of wholeness while focussed on the physical world nevertheless marks out the main contours of meaning to a remarkable degree. However, by focussing on the physical world certain terms he uses, such as ‘order’ when used to refer to the implicate and explicate orders masks to some extent the implication that these are the orders of meaning. In general, our holistic theory of meaning is entirely inclusive, non-Cartesian and that means while inclusive of the local human mind it also has a focus on universal consciousness, which in another place we have called the nonlocal [7].

In this paper, we would stress that we are not offering a radical idealist interpretation of Bohm and Hiley’s work. Radical idealism is the metaphysical view that is associated with ideas in the mind. While we do present a metaphysical view here, the reality of meaning is not directly associated with ideas, but with the self-know experiences of meaning exchanges and meaning making. These actions are always prior to the formation of concepts and ideas [8,9]. In addition, we are not attempting to ‘put words into Bohm mouth’ but simply endeavoring to excavate the full implications of what he and Hiley have written.

Our approach to meaning is of an omnipresent reality and far from viewing it as a by-product of signs, language, mind or intentionality, (the conventional views) our approach is in line with Bohm’s proposal, that meaning represents the first and foundation agency of the universe. We could also add that any act of making meaning represents the self-known experience of intelligibility that involves both implicit qualities and explicit quantities. As a First Order experience meaning precedes thought, ideas, concepts, expressions as well as the inorganic physical world and also organic forms of life for all these are but derivative manifestations of Bohm’s derivative explicate order.

It is unnecessary here to detail every feature of our theory of meaning, however, suffice to say that its general lines follow closely [with some exceptions) to Bohm’s approach to wholeness and his theory of the implicate and explicate order. Bohm and Hiley were both physicists who often referred to meaning in general terms but neither of them crossed the bridge into analyzing meaning as a subject matter and as a consequence, neither of them had any analysis of meaning’s structure or function. As physicists, their emphasis always tended to be on the physical features of wholeness and away from the underlying metaphysical strata of wholeness. Yet the metaphysical foundations of the physical world are always there in Bohm’s theory as the terms, ‘enfolding’ and ‘unfolding’ testify.

In the late 1980s Bohm authored a book on meaning, called *Unfolding Meaning: A Weekend of Dialogue with David Bohm* [10]. In it, he directed a group discussion toward the fascinating question of how our meanings relate to the universe as a whole. Bohm had a persistent concern about the relationship between mind and matter and in these discussions, he began to investigate the relationship between three crucial features: matter, energy and meaning. With this discussion he was suggesting that matter, energy and meaning may have similar fundamental roles to play in the universe.

Actually, he goes further than that because he was suggesting that meaning had an agency that could enfold both matter and energy and then concludes that meaning represents the more fundamental state than either energy or matter. This is perhaps an extraordinary statement for an internationally acclaimed physicist to make because meaning is not a physical state but fits the requirements of a prior metaphysical state. Hence, the implications of Bohm’s comments in relation to matter, energy and meaning are quite radical in that the so called independent solid, physical world that physicists talk about in terms of energy and matter may not be the primary foundation after all but rather, as Bohm suggested is enfolded within a metaphysical world of meaning, which is more fundamental.

Bohm reasoning in this conclusion is that the relationship between energy, matter and meaning is not equal because meanings can enfold meanings, but they can also enfold matter and energy. Hence: ‘Matter enfolds energy, and energy enfolds matter’, however, energy cannot enfold energy and matter cannot enfold matter’. Following this logic, Bohm concludes that while ‘meaning refers to itself directly, and this is in fact the basis of the possibility of that intelligence which can comprehend the whole, including itself. On the other hand, matter and energy obtain their self-reference only indirectly, firstly through meaning’. [10, p.91] In another reference, Bohm writes the supporting comment that ‘the cosmos may be ordered according to a kind of “objective” meaning.’(11, p.180) Here is direct evidence that after the development and publication of this theory of wholeness and the implicate order Bohm insists that meaning (a metaphysical state) is more fundamental than either the physics of energy or matter.

Bohm’s reference to ‘an intelligence that can comprehend the whole, including itself’ is also evidence of the further step he takes in relation to meaning and that was that meaning is the essential nature of consciousness. [12, p.436] In *Unfolding Meaning* he gives support to that contention by writing, ‘Any fundamental change in meaning is a change in being for us. Therefore, any transformation of consciousness must be a transformation of meaning’. [10, p.93] He also writes that ‘We can say that human meanings make a contribution to the cosmos, but we can also say that the cosmos may be ordered according to a kind of ‘objective’ meaning’. [10, p.97] And again, ‘I think conscious awareness, its essential feature, is meaning.’ … ‘The activity of consciousness is determined by meaning’. [10, p.102]

Thus, in this book when referring to meaning and in particular to ‘objective meaning’ he is implying a universe of intelligence – and by linking conscious awareness to meaning, this becomes – universal consciousness. Supporting that conclusion is his suggestion that meaning had agency that can enfold both matter and energy along with his conclusion that meaning is more fundamental than either energy or matter. In addition, Bohm states that, ‘Meaning organizes everything’ [12, p.443] and again, ‘meaning is the essence of reality’ [12, p.441]. Hence, with these statements Bohm positions meaning as the fundamental ground of the universe. While he did not actually write the words, *meaning represents the content of consciousness* the implication that follows from his proposition concerning the relationship between meaning, which organizes everything and consciousness, leads directly to this conclusion, that meaning as the content of universal consciousness has the agency that can organize everything.

The implications of this conclusion are extensive. Take one small example from biology, in relation to the cell-centered view of evolution the linking of meaning and consciousness is significant, especially in relation to the question of the content of cell-to-cell communication [13]. In their paper ‘The Cosmologic continuum from physics to consciousness’, Torday and Millar write, ‘Life is dependent on information that is communicated between the cell and its environment, or between cells’ [4]. We would respectfully suggest that all communication, whether between cells or their environment or even between people, represent exchanges of meaning, rather than ‘information’. ‘Information’ represents a discursive artifact of the current mechanic/technological, Cartesian sub-culture, which is a cultural context that we doubt has much relevance to the life of cells or to cell-to-cell communication. The issue of ‘meaning’ rather than ‘information’ is critical to how communication is generally understood whether as part of a Cartesian order suitable for classical mechanics, or as an integrated movement within the wholeness of the holomovement.^1^

Inherent in Bohm linking of meaning and consciousness is the related question of the content of consciousness that is associated with acts of communications. If meaning does represent the content of consciousness as Bohm’s work implies, then meaning must also represent the content of communication. Such implications lead directly away from the current preoccupation in physics, technology and biology with the divisive term ‘information’ and back into a metaphysical engagement that the term meaning suggests. While the statement: *meaning represents the content of consciousness* may seem a radical proposition to assert, we suggest that it simply presents a challenge to any Cartesian researcher to find some meaning that is devoid of consciousness, or the reverse. We believe that such a demonstration would be impossible. Hence, Bohm’s conclusion in these documents, that meaning/consciousness are two side of the same coin is highly significant for the whole of science and that includes our understanding of evolution.

This linkage of meaning and consciousness tells us that the context of the holomovement is that of consciousness and as the holomovement refers to everything in the universe it means that the context of the holomovement is universal consciousness. As a consequence, the universal context of the holomovement would then be entirely filled with the content and the conditions of meaning. Such a conclusion gives a coherent and meaningful depth to the holomovement. However, we should point out that neither Bohm or Hiley explicitly stated that the implicate order and its enlarged potential, the holomovement represented the context of universal consciousness, but that conclusion strongly presents itself once we follow through on the implications of what both authors have stated.

Bohm’s thinking on the subject of consciousness oscillated around several positions and often slides seamlessly from one position to another. At times, he advocated a panpsychist view as seen (above) in his references to meaning, energy and matter. At other times, he took a dual-aspect position, – ‘each level of the unbroken whole of reality there will be a ‘mental pole’ and a ‘physical pole”. [5, p, 285] Finally, he and Hiley were not averse to relying upon a more mainstream Cartesian reductionist position, like the following taken from Bohm and Hiley’s highly original 1995 book, *The Undivided Universe*: ‘Throughout this book it has been our position that the quantum theory itself can be understood without bringing in consciousness and that as far as research in physics is concerned, at least in the present period, this is probably the best approach’. [6, p.381]

In order to get some stability in our understanding of Bohm’s view of consciousness it is necessary to look more closely at some of the implications of what he and Hiley have written in regard to wholeness, consciousness and meaning.

## Implications

Returning to the genesis of Bohm’s implicate order, that of implicit meaning, in terms of our theory of meaning (which closely follows Bohm’s hierarchy) the term ‘implicit’ represents the larger nonlocal aspect of meaning while the smaller local aspect is that of explicit meaning. By focussing directly on the nature of meaning, we arrive at an understanding that implicit meaning has more qualities than the transformations that underpin the processes Bohm called enfoldment and unfoldment. These are conditions that represent how meaning re-organizes itself when implicit meaning transforms itself into explicit meaning and again, when explicit meaning is transformed back into implicit meaning. A common example of this is in thought. Different thoughts arise out of a background of implicitness (they unfold) and persist for a while and then disappear after a time, to be enfolded back into the implicit background of consciousness.

Of the many qualities of implicit meaning, at the forefront are the tacit interconnections that establish background contexts. Contexts provide the necessary unity that underpins and organizes every system. The necessity of underlying interconnections and unity that organizes every systems is pertinent to the controversies in evolutionary biology where traditional Darwinian competition-oriented evolution has been contrasted to symbiotic and cooperation theories. The evolutionary biologist Lynn Margulis (1938–2011) has been the primary proponent of the theory of evolution through symbiosis. Considered a radical by her traditional peers, Margulis’ endosymbiotic theory reflects the natural underlying unity of implicit meaning and by extension, the unifying role of Bohm’s holomovement. Her approach to evolution has been summed up by Torday who wrote that Lynn Margulis, ‘dictates that we are ‘of’ this Universe’ rather than ‘in’ it [14].

From the wholeness perspective of meaning, the controversy over evolution by competition or cooperation points to the different worldviews that rest on the value we place on the implicate or explicate orders, that is, on implicit or explicit meaning [9]. Traditional mainstream science has tended to employ Cartesian views that assume a world of separation and division, which is the natural result of an exclusive focus on the differences of the explicate order, that is, of maintaining an exclusive focus on explicit meaning. In relation to evolution, this locally oriented and deterministic worldview will emphasize competition simply because the underlying unity of all systems has been ignored or erased from this worldview and so competition (in the form of competing differences) is then seen as the natural form of interaction between organisms. In contrast, an evolutionary theory that implies an underlying organizing and unifying context (of implicit meaning) will result in the kind of emphasis Margulis gives to symbiosis and its crucial role in symbiogenesis, that is, in organisms living together. As Bohm’s implicate and explicate orders are not equal (the explicate always arises out of the implicate) so a theory of evolution that rests on the underlying nonlocal unity of implicit meaning must be marked as superior to a local and deterministic theory that reflects and over-values the secondary attributes (explicit differences) of the explicate order.

Returning to the several qualities of implicit meaning, each of the three qualities mentioned (context, unity and interconnection) add meaning to Bohm’s model of the implicate and explicate orders by connecting the parts to the totality of the holomovement. Hiley tell us in his 2008 paper that ‘the importance of context in quantum theory has only recently begun to emerge. However, in the Bohm interpretation context dependence becomes crucial’. [1, p.15] Hiley goes further stating that contextual dependence is vital not just to quantum mechanics but to other areas of human activity including especially philosophy and psychology [1, p.22]. We agree that context dependence is not only crucial to quantum theory but also for every area of analysis and that includes Bohm’s model of wholeness. Hence, the background context that the holomovement represents acts by unifying through complex and implicit interconnections the totality of its individual parts.

Perhaps the most pertinent quality of implicit meaning to this discussion is implication. Implications represent the multiple possibilities that arise from every explicit action, behavior or expressions and, therefore, these possibilities represent the future consequences and potential paths that need to be accounted for, resisted or pursued. The implications embedded within a theory are crucial for a fuller comprehension of the theory and this is the case with regard to the implications within Bohm’s model of the holomovement and his view of consciousness. In this respect we need to ask, what was Bohm and Hiley referring to when they write that the background from which all physical phenomena arise is the holomovement? Is the holomovement an idea, an abstract principle or universal consciousness?

In his 2008 paper, Hiley writes that ‘the word “movement” invariably invokes the response “movement of what?” But in our terms, movement or process cannot be further analyses’. [1, p.10] His justification for this lack of analysis is that movement or process is a primitive description from which all else follows but suggests it can also replace the term ‘field’ as a primitive description of present-day physics. In addition, following the philosophy of Whitehead (1939) Hiley’s preference is for the word ‘process’ rather than ‘movement. He writes, ‘What I have tried to suggest here is that by using the notion of process and its description by an algebraic structure, we have the beginnings of a descriptive form that will enable us to explore the relations between mind and matter in new ways’. [1, p.21] However, we would suggest that the word ‘process’ is equally open to the question ‘process of what’? As is the terms ‘movement’, ‘field’ or ‘order’.

An answer to the ‘what’ question in each of these cases will provide us with the important contextual details of these processes so we then will have a direction and focus for further analysis, discussion and understanding. However, if we choose to remain content with the orphan terms ‘movement’ or ‘process’ or ‘order’ this inevitably will mean we are content to leave their contexts void of content and conditions. As serious researches this is not the position we can accept as we have already agreed with the implications in Hiley’s comments that context is crucial. Contexts are always crucial and especially when it comes to the wholeness of Bohm’s model of the holomovement.

In order to discover the conditions of the content of this universal context and thus its fuller meaning we suggest the need to go back and relook at implicit meaning because one of the major qualities of implicit meaning is context. Implicit meaning comes in contexts and, therefore, the content of every context will be implicit meaning. The ‘what’ question in regard to ‘movement or ‘process’ is then answered by the conclusion that the movement within the holomovement is that of implicit meaning. How Bohm would respond to this kind of conclusion is ambiguous. For example, his treatment of the holomovement as fundamental has presented mainstream reductionist science with a serious difficulty because this primary state he writes is ‘undefinable and immeasurable’ in its ‘unbroken and undivided totality’. [11, p.131]

Bohm states that its law cannot be stated, for the ‘total law of the undefinable and immeasurable holomovement could never be known or specified or put into words. Rather, such a law has necessarily to be regarded as implicit’. [11, p.137] This last reference to ‘implicit’ represents an excellent description of the content of the holomovement, as we have suggested, the holomovement is entirely full of implicit meaning. However, is implicit meaning inherently ‘undefinable and immeasurable’? In other words, are there any intrinsic conditions of the implicitness of the holomovement able to be identified and described? Neither Bohm or Hiley answer such questions as these, however, Bohm modifies his comment regarding ‘undefinable and immeasurable’ when he writes that the law of the primary and fundamental holomovement represents an ‘immense multidimensional ground.’ [11, p.118] In terms of meaning, we would suggest that the implicit content of the holomovement cannot be measured but it does allow for a surprising range of multidimensional descriptions.

Bohm has also stated that the holomovement is ‘life implicit’ and it represents the ground of ‘life explicit’, [11, p.102] yet throughout his work he does not focus on these foundational terms of ‘implicit’ and ‘explicit’ meaning, rather, he works with their derivations (implicate order and explicate order). This path has a tendency to situate consciousness as a local and private feature of the individual. For example, the primary quality of Bohm’s holomovement he says is ‘self-existent and universal’ [11, p.102] and which ‘applies both to matter (living and nonliving) and to consciousness’. [11, p.104] In these statements the holomovement appears not to be conceived of as universal consciousness, even though he says it is ‘life implicit’.

We suggest that if life and agency are general features of universal consciousness, then ‘life explicit’ will represent organic life forms such as birds and animals and human being while ‘life implicit’ will represent the general form, which is ‘life’ itself. If the holomovement contains both the potentials of ‘life implicit’ as well as the capacity to produce the life cycles of transient life forms then this discussion has moved the holomovement from physics into the areas of biology and the life sciences, (where it intersects with the organistic or holistic philosophy of nature suggested by Gilbert, S. F., and Sarkar, S., 2000).^2^ Yet these kinds of interconnections related to universal consciousness have arisen naturally from Bohm and Hiley’s statements that were not pursued, although Hiley does identify “Being” as the outward manifestation of becoming and discusses the development of a mathematical formulation to describe this process. [1, p.10.] Perhaps the reason for this lack of extended discussion is the containment of consciousness to the local level where like matter it is treated as one of the holomovement’s local features.

A further comment on how Bohm viewed consciousness is when he attributes implicit and explicit qualities to consciousness and says, ‘Whatever may be the nature of these inward depths of consciousness, they are the very ground, both of the explicit content and that content which is usually called implicit.’ [11, p.117] Again, when referring to the actual structure and function of thoughts, ‘We see then, that each moment of consciousness has a certain explicit content, which is a foreground, and an implicit content, which is a corresponding background’. [11, p.111] And then, ‘Consciousness is possibly a more subtle form of matter.’ [11, p.148] While this last comment is ambiguous in relation to the Cartesian view, none of these references locate consciousness as universal but they leave the firm impression that Bohm’s use of ‘consciousness’ represented a quality of the local human mind. If that is the case, then this view is not consistent with his comments in *Unfolding Meaning* where he links meaning to a consciousness that can comprehend the whole, including itself, and then when he proposes that meaning is more fundamental than energy or matter.

We would argue that in order to come to some informed consideration concerning the wholeness of Bohm’s holomovement it is necessary to begin by asking about the conditions of implicit and explicit *meaning*, rather than relying upon his secondary terms of the implicate and explicate orders. It is also necessary to pursue the many oscillating implications within Bohm and Hiley’s work in order to pin them down to the scope of their use of the term ‘consciousness’ and the relationship that term has to meaning. For example, how are we to interpret these comments when Bohm states that ‘meaning is fundamental to what life actually *is*’ [11, p.180] and that ‘the universe is its meaning’ [11, p.181] or again, ‘there is no point in asking the meaning of life, as life *is* its meaning’, and again, ‘not only that there is a meaning to it, [the universe as a whole] but rather *that it is* meaning’ [12, p.438.]? From Bohm’s oscillating views on consciousness, they can represent ambiguous statements. However, from the standpoint presented here that meaning is the fundamental ground of universal consciousness Bohm’s comments are coherent in that they reinforce the contention that *the holomovement as ‘life implicit’ is universal consciousness*. As such the holomovement will contain the agency and the transformational potentials to produce the life cycles of diverse, transient life forms.

Hiley writes, ‘if we put wholeness centre stage then Nature at its very core is organic and by using the term organic, I am using it in the same spirit as Whitehead (1939)’. Then he goes on, ‘in this view the atoms, molecules, fields and ultimately space-time itself arises from activity, process. By starting from this more basic position we hope to lift the veil of reality further’. [1, p.7–8] We suggest that when the Cartesian veils are fully lifted from Bohm’s holomovement we will see clearly that the First Principle of physics, biology, physiology, mathematics, psychology and society at large is the unity and interconnection of universal consciousness, which operates as a holomovement in regard to every implicit and explicit exchange or transformation. Such a clear view will mean that Nature at its very core is organic, sentient and rich with meaning and hence, the universe is not dead but within its living heart is intelligent, organic and full of meaning.

## Notes

1. Both Bohm and Hiley have used the term ‘active information’ to discuss the exchanges of the implicate and explicate orders, however, we would respectfully suggest that such terminology is detrimental to the wholeness worldview. Bohm’s comment that ‘information is a condensed form of meaning’ [12, p.442] introduces an un-necessary Cartesian gap between meaning and information that is unable to be bridged. For a more complete discussion see: Lohrey, A.,‘The Language Virus of Information Theory’, Edgescience, #35, September, 2018/5.

2. “Embracing Complexity: Organicism for the 21st Century” *Develop Dynam* 219: 1–9).
